# Critical appraisal of “Colchicine for the secondary prevention of cardiovascular diseases: a cumulative-dose meta-analysis of randomized controlled trials including 31,397 subjects worldwide”

**DOI:** 10.1097/MS9.0000000000004430

**Published:** 2025-12-03

**Authors:** Asra Amjad, Muhammad Junaid, Umair Ali, Muddassir Khalid

**Affiliations:** a Department of Medicine, Islamic International Medical College Rawalpindi Pakistan (Riphah International University), Islamabad, Pakistan; b Department of Medicine, Lady Reading Hospital, Peshawar, Pakistan; c Department of Pharmacy, University of Swabi, Swabi, Pakistan; d Department of Medicine, Nishtar Medical University, Multan, Pakistan

*To the Editor,*

Li *et al*’s new meta-analysis on cumulative colchicine dose and cardiovascular outcomes caught our attention^[1]^. Although this study provides helpful and instructive data, some methodological issues should be examined more closely since they might have increased the explored advantages of colchicine in secondary prevention. The suggested ≥90 mg-day barrier is an ecological fallacy and is a study-level construct rather than a patient-level biology. Genetic background, renal clearance, and adherence all influence pharmacokinetic variance. An individual participant data (IPD) study with exposure-response modeling is considerably more appropriate to account for drug exposure, renal state, and adherence because of these person-level intricacies, which make an aggregate cutoff risk oversimplification^[2]^.

For occurrences that were reported as time-to-event outcomes, the study combined odds ratios. When dangers change over time, odds ratios based on total counts may skew the results. More trustworthy metrics, like hazard ratios or restricted mean survival time, are necessary in these situations to produce accurate estimates and guarantee clinical interpretability^[3]^. Although a decrease in C-reactive protein was mentioned as proof, even slight adjustments to one biomarker are insufficient to demonstrate a therapeutic effect. In order to connect biological effect with results, previous anti-inflammatory trials have demonstrated the necessity of a multi-marker inflammatory profile, including NLRP3 activation, neutrophil–lymphocyte ratio, and interleukin (IL)-1β activity^[4]^. This emphasizes that complete immune signals, not isolated surrogates, are the foundation of biological plausibility.

A misleading sense of uniform efficacy is created when diverse trial contexts, such as post-myocardial infarction, percutaneous coronary intervention, chronic coronary artery disease, and stroke populations, are pooled. Crucially, CLEAR-SYNERGY (Colchicine and Spironolactone in Patients with Acute Myocardial Infarction/SYNERGY Stent Registry), the biggest acute MI study, showed no benefit and ought to be given the proper weight in sensitivity analysis^[5]^. Conclusions run the risk of overstating generalizability across extremely diverse populations if subtle weighting is not used. Clinically significant toxicities such as myopathy, marrow suppression, and gastrointestinal intolerance were not sufficiently addressed by the safety assessment, which was restricted to death. When statins, Cytochrome P450 3A4 (CYP3A4) or P-glycoprotein inhibitors, and compromised renal function are present, these risks are increased. Therefore, a stratified safety study that takes into account medication interactions would give physicians a more accurate assessment of colchicine’s acceptability^[6,7]^. Lastly, there is a risk of overgeneralization when a generalization of effectiveness across several ethnic groups without pharmacogenomic stratification is made. Ancestry-stratified IPD investigations are required to address the issue of altered colchicine handling, which is known to be caused by ABCB1(ATP-Binding Cassette Subfamily B Member 1) polymorphisms and associated variants^[8]^. Confidence in the pooled effect is further limited by moderate heterogeneity, trial-level inconsistency in MACE (Major Adverse Cardiovascular Events) definitions, and the limitation to English-language trials. To lessen bias and boost robustness, future synthesis might benefit from trial sequence analysis, core outcome sets, and the methodical inclusion of non-English randomized controlled trials^[9]^. Lastly, although Li *et al*’s study offers valuable information, its technique may overlook the exaggerated advantages of colchicine. A more transparent and more trustworthy image would be obtained with a more detailed method that includes multi-marker inflammatory profiling, hazard ratio modeling, IPD analysis, and better safety evaluation. To improve the clinical usage of colchicine, more research is required to address trial heterogeneity, medication interactions, and ethnic diversity.

This letter to the editor complies with the TITAN guideline^[10]^.

## Data Availability

Not applicable.
